# Stability of commercial parenteral lipid emulsions repacking to polypropylene syringes

**DOI:** 10.1371/journal.pone.0214451

**Published:** 2019-04-10

**Authors:** Dorota Watrobska-Swietlikowska

**Affiliations:** Department of Pharmaceutical Technology, Medical University of Gdansk, Gdansk, Poland; University of British Columbia, CANADA

## Abstract

To accommodate small fluid volumes, repackaging of intravenous lipid emulsions is frequently performed in hospitals providing parenteral nutrition to neonates and smaller pediatric patients. The physical stability of lipid commercial parenteral emulsions repacked and stored in polypropylene syringe up to 30 days at room temperature, refrigerator and 40°C was determined to establish options for extended storage. Lipid emulsions in the manufacturers’ original containers were used as references. Commercial lipid emulsions (20% of oil phase), ClinOleic, Intralipid, Smoflipid, Omegaven and Lipofindin LCT/MCT were repackaged under aseptic conditions in polypropylene syringes and stored at 4°C, 25°C and 40°C without light protection. Samples were assayed periodically over 30 days using validated, stability-indicating methods. Lipid emulsions in the manufacturers’ containers stored in the same conditions were as references. Analysis of variance showed differences in the physical parameters due to temperature (p<0.05) and study day (p<0.05) but not the type of the emulsion (p = 0.98). The parenteral lipid emulsions in polypropylene syringe exhibited identical (except Z-avarage at 40°C, t = 30 days) to original containers time-dependent behavior taking into account the mean globule size, pH, and zeta potential measurements. Size of oily droplets of all test conditions remained below the United States Pharmacopeia limits. The results allow safe repacking of commercial lipid emulsion in a syringe, which is a necessary condition for supplying parenteral nutrition using the two-in-one method for newborns. However, longer storage than 12 h of repacked emulsion needs microbiological studies.

## Introduction

The beyond-use date for intravenous medications following reconstitution or repacking is often limited because of the potential for breaks in sterility and physicochemical stability in the new container (interaction with the container’s surface). However, when reconstitution and repacking are carried out in a sterile environment, following USP Chapter <797> recommendations [1] it is entirely reasonable to extend the beyond-use dates of these products beyond 12 h (microbiological stability). Extending the beyond-use date may reduce the physicochemical stability of parenteral emulsions and, consequently, results in the safe administration of such mixtures to patients.

Hospital care for newborns, especially those with a with low birth weight, is very complicated due to the fact that their brain and organisms develop very quickly and need much more nutrients. Parenteral nutrition is needed in these small patients because most cannot meet the majority of their nutritional needs using the enteral route. Despite the adoption of a more aggressive approach with amino acid infusions, there is still a reluctance to use early intravenous lipids [2]. Due to the high risk of incompatibility between lipids and other parenteral nutrition components, lipid emulsion must be administered separately from the parenteral nutrition solution in neonates. The acidic pH of a parenteral nutrition solution is necessary for maximum solubility of calcium and phosphorus. If a lipid emulsion is added to the parenteral nutrition solution, as is done in 3‐in‐1 (total nutrient admixture) solutions, the high amount of calcium and phosphorus needed by these infants may result in an unseen precipitate with serious consequences. Continuous fat infusion over 24 hours is the preferred method in neonates [3]. For this reason, commercial lipid emulsions are repacked to a polypropylene syringe from original commercial bags and administered intravenously to neonates at neonates wards with a higher temperature [4]. The higher temperature in neonatal wards could be an additional factor negatively affecting the stability of lipid emulsion. There is very little information about the stability of lipid emulsion repacked to polypropylene syringe and stored at a higher temperature.

Storage of lipid emulsion in plastic containers is controversial. It was shown that patients who received lipids delivered in plastic bags are more likely to have hypertriglyceridemia than those who received lipids from glass bottles [2]. This is possible because of a higher proportion of large-diameter fat globules in plastic bags. Another aspect is the stability of lipid emulsion in plastic bags. Most commercial lipid emulsions are usually packed in type I glass bottles, however, only one ClinOleic emulsion is produced in a plastic bag. Plastic containers are permeable to oxygen and contain oil-soluble plasticizers and are thus usually avoided [5]. Several studies have shown that lipid injectable emulsions stored in plastic containers exceed the PFAT5 limits for the size of oil droplets (PFAT5) proposed by USP <729>, making plastic containers less suitable than glass containers [6–8]. However, manufacturers still try to improve the stability of lipid emulsions in plastic containers [9]. In other studies, it was suggested that plastic containers could decrease the PFAT5 because ethylene vinyl acetate (EVA) and polyvinylchloride (PVC) containers can function as a sink for the emulsion globule triglycerides and the larger globule sizes have a greater relative affinity for adsorption compared to the smaller sizes [8]. Despite the fact that storage of lipid emulsion in plastic containers is necessary in clinical practice, in terms of physicochemical stability it is problematic.

Lipid emulsions consisting of soybean oil and lecithin were originally intended to provide essential fatty acids and energy. Today they are used as carriers of lipophilic drugs, especially vitamins A, D, E, administered parenterally [10]. The droplet size of lipid emulsion can have a direct impact on the toxicity and stability of this system. An increase in the droplet size is the first indication of formulation stability issues. Moreover, droplets greater than 5 μm can be trapped in the lungs and cause pulmonary embolism. Pulmonary embolism may develop and is associated with a high risk of morbidity and mortality [11]. The second aspect is that large globules (≥5 μm) exceed the diameter of capillaries are postulated to become trapped within these vessels. The result of such embolic occlusions is hypothesized to be tissue injury (i.e., necrosis), inflammation, and compromised organ function [12]. Many physiochemical parameters such as optical microscopic observation, pH and zeta potential measurements should be monitored to verify the stability of parenteral emulsion. However, the droplet size and distribution are amongst the most important characteristics of a lipid injectable emulsion [5]. The stability of lipid globules is a critical parameter that needs special analytical methods and equipment. Neither European nor Polish Pharmacopeias propose droplet size limits for lipid injectable emulsion. However, two limits for the globule size distribution are provided by the United States Pharmacopeia (USP): mean droplet size (MDS) of the globules, which should not exceed 500 nm and the second one regards the percentage of the volume of the large-in-diameter tail of the lipid droplet distribution related to the total lipid volume (PFAT5), which should not exceed 0.05%. It recommends two methods for particle size determination: Method I and Method II. Method I involves light-scattering techniques that are used to determine MDS. For measurement of mean droplet size, the use of either dynamic light scattering also known as photon correlation spectroscopy or classical light scattering based on Mie scattering theory is recommended. Method II is a light obscuration or light extinction which employs a technique named single-particle optical sizing (SPOS) and is based on the effect produced by a particle as it crosses a beam of laser light and used to determine PFTA5. Method II allows determining the extent of the large diameter droplet tail (> 5 μm).

The objective of this study was to evaluate the physical stability of five of the most commonly used commercial lipid emulsions: Intralipid 20%, Lipofundin MCT/LCT, Omegaven, SMOFlipid and ClinOleic 20% repacked to polypropylene syringes and stored at 4°C, room temperature (25°C) and 40°C for 30 days. This study is useful in the clinical practice of parenteral nutrition in neonates and is a response to many questions from hospital pharmacists. Lipid injectable emulsions in the manufacturers’ original containers, stored in the same conditions, were used as references. During the 30-days study period, a physical analysis was performed at 4 time points (days 0, 7, 14, and 30). On each study day, each emulsion was inspected visually and microscopic observations, pH and zeta potential measurements as well as globule size distribution were performed.

## Materials and methods

### Repacking of emulsions to polypropylene syringes

Five of the most commonly used commercial lipid injectable emulsions: ClinOleic 20%, Intralipid 20%, Lipofundin MCT/LCT, Omegaven and SMOFlipid were repacked to polypropylene syringes under aseptic conditions. Emulsions in the manufacturers’ containers were used as references. Emulsions in original containers as well as in the syringes were stored in climatic chambers at 25°C/60% rH, 40°C/75% rH and in a refrigerated cabinet at 4°C±1°C. The study was carried out up to 30 days to determine the point of destabilization. Analytical measurements described below were carried out directly after preparation (t = 0) and after 7, 14 and 30 days of storage. Each sample was prepared in triplicate.

### Physical analysis

Emulsions were subjected to a physical stability analysis consisting of a visual inspection, a microscopic observation (biologic microscope with camera B1 223A *Motic*, Wetzlar, Germany), determination of oil globules size distribution–laser diffractometer (MasterSizer E *Malvern Instruments*, Malvern, UK) and photon correlation spectroscopy (Zetasizer, *Malvern Instruments*, Malvern, UK), zeta potential (Zetasizer, *Malvern Instruments*, Malvern, UK), pH measurement (pH meter *Orion 350*, Beverly, USA, with combination electrode).

#### pH measurement

Before each pH measurement, a two-point calibration of the pH meter was performed, each with a buffer solution of pH 9.00 and pH 4.00, respectively. The pH 7.00 solution was used afterwards as a control. Between the calibration steps, the electrode was rinsed with distilled water and wiped dry. Each sample was measured in triplicate after 5 min of equilibration, so n was 9.

#### Microscopic observation

The physical stability of emulsions was assessed by a lipid droplet measured in a light microscope with an upper droplet size of ≥1 μm. Each microscopic sample (10 μL by a manual pipette) was analyzed with 40-fold magnification. Ten individual visual fields were inspected per microscopic sample (30 total visual fields/aliquot): five in the corner and five in the middle of the preparation. The size of the lipid droplets in the visual field was determined using an ocular micrometer (0.01 mm). The diameter of the largest lipid droplet was measured and counted in each of the 15 visual fields tested per aliquot. The diameter of the largest lipid droplet and the number of lipid droplets above 5 μm were measured and counted in each of the 15 visual fields tested per aliquot.

#### Size of oil droplets

The droplet size of emulsions was determined using dynamic light scattering (DLS), which covers a size range of 20 to 5000 nm and uses a helium-neon laser light and integrated analysis software (Zetasizer Nano ZS model ZEN 3600, Malvern Instruments, Malvern UK). Each sample was determined in triplicate at 21°C (n = 9 for each emulsion and each point of analysis). Data are shown in terms of the effective mean diameter (Z-average) and the polydispersity index (PI), which reflects the width of the particles droplet size distribution. The samples, 1 mL, were collected from each emulsion, under a laminar flow hood, with sterile syringes and needles and diluted with water for injection 1:100. Each analysis was performed in triplicate (n = 9 for each emulsion).

The second technique was laser diffractometry (MasterSizer E, Malvern Instruments, Malvern, UK). All the results were calculated according to the Mie theory. The chosen data analysis method was the monomodal method set. 1 mL of sample was collected from each emulsion with sterile syringes and needles and directly transferred to 500 mL of injection water for a tank equipped with a stirrer. Data were transferred from Mastersizer software for calculation of the volume diameters D50, D90 and Dmax which means 50% and 90% or all of the particles are below the given size. Each analysis was performed in triplicate (n = 9 for each emulsion).

#### Zeta potential analysis

Zeta potential was determined by microelectrophoresis using a Zetasizer Nano ZS (Malvern Instruments, United Kingdom). An electric field was applied to the admixtures and lipid globules would then move with a velocity related to their zeta potential. The Zetasizer measured the velocity using a patented laser interferometric technique called M3-PALS (Phase Analysis Light Scattering). This enables the calculation of electrophoretic mobility, and from this, the zeta potential for an accurate measurement using the Smoluchowsky formula and the results are expressed in mV. Measurements were carried out at 23 ± 1°C. Each PN admixture was analyzed in triplicate (n = 9 for each composition), so n for each composition was nine. Samples were diluted 1:100 with water for injection water and injected with 2 mL syringes into the microelectrophoretic cell. Between each measurement, the cell was rinsed with water for injection.

### Statistical analysis

The results are presented as means ± standard deviation (SD). All measurements were made in triplicate unless stated otherwise. The data were analysed using GraphPad Prism 5.0 (GraphPad Software, San Diego, CA, USA). For multiple comparisons, we used a one-way ANOVA, followed by the Newman-Keuls multiple comparison test. The priori level of significance was 0.05.

## Results

### Visual observations

Regardless of the type, in each of the commercial lipid injectable emulsions tested in polypropylene syringes (ClinOleic, Intralipid, Lipofundin MCT/LCT, Omegaven and SMOFlipid) no visual changes were found. No changes in the color or phase separation over time were observed, regardless of the storage temperature of the emulsion. The results were referenced to emulsions in original containers.

### Microscopic observations

**Fig 1:**
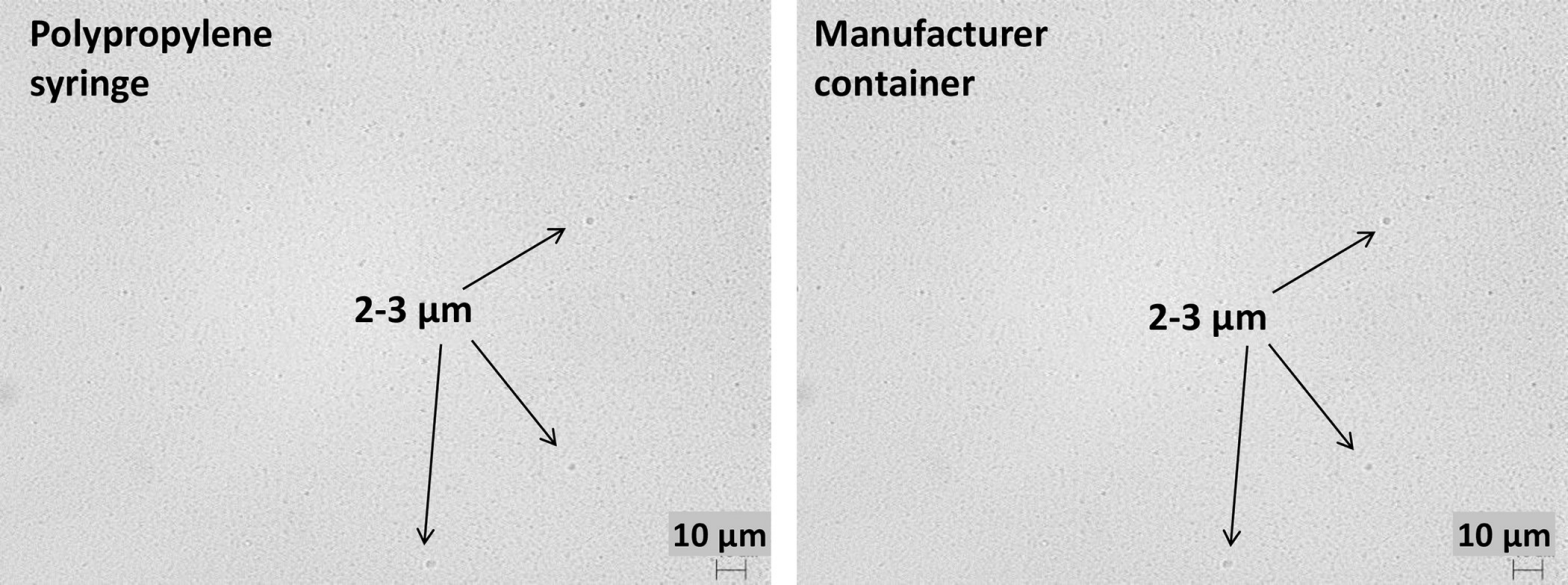
Microscopic observation of SMOFlipid in polypropylene and original containers (t = 30 days, 40°C) (scale 10 μm).

Lipid injectable emulsions in polypropylene syringes in the microscope image were characterized by drops of the oil phase with a size below 2 μm and the presence of 2–3 μm of oil droplets. No change in the size of oil droplets was noticed regardless of the type of emulsion. In all emulsions after 30 days at 40°C, droplets of 2–3 μm were observed (Table 1, Fig 1).

**Table 1:** Emulsions in polypropylene syringes–the influence of time and temperature of storage (n = 9; mean ± SD; p< 0.05).

Time [days]	Temp [°C]	Size of oily droplets					pH	Zeta potential [mV]	Microscopic observation
		Z-average [nm]	pDI	d_(0.5)_ [nm]	d_(0.9)_ [nm]	d_max_ [μm]			
**Omegaven**									
**0**	**4**	**223 ± 0.85**	**0.062 ± 0.034**	**290 ± 0.000**	**510 ± 0.005**	**1.00**	**7.98 ± 0.01**	**-43 ± 0.49**	**< 2 μm**
7	4	219 ± 1.84	0.073 ± 0.012	280 ± 0.005	530 ± 0.036	1.00	7.85 ± 0.01	-47 ± 0.70	< 2 μm
	25	213 ± 1.76	0.066 ± 0.015	280 ± 0.005	530 ± 0.023	1.00	7.89 ± 0.02	-48 ± 0.11	2–3 μm
	40	213 ± 1.55	0.080 ± 0.028	280 ± 0.005	520 ± 0.011	1.00	7.83 ± 0.02	-44 ± 2.80	2–3 μm
14	4	213 ± 1.00	0.075 ± 0.020	280 ± 0.005	520 ± 0.011	1.00	7.84 ± 0.01	-44 ± 0.30	< 2 μm
	25	212 ± 0.66	0.060 ± 0.025	280 ± 0.010	510 ± 0.025	1.00	7.72 ± 0.00	-45 ± 0.75	< 2 μm
	40	217 ± 2.18	0.065 ± 0.036	290 ± 0.005	510 ± 0.005	1.00	7.72 ± 0.00	-49 ± 0.66	2–3 μm
30	4	215 ± 2.35	0.060 ± 0.007	290 ± 0.000	530 ± 0.017	1.00	7.80 ± 0.01	-41 ± 0.52	< 2 μm
	25	213 ± 4.44	0.055 ± 0.012	300 ± 0.005	520 ± 0.020	1.00	7.68 ± 0.01	-42 ± 0.30	< 2 μm
	40	203 ± 0.43	0.084 ± 0.007	270 ± 0.011	470 ± 0.050	1.00	7.30 ± 0.00	-38 ± 0.95	3–4 μm
**Intralipid**									
**0**	**4**	**281 ± 2.53**	**0.090 ± 0.020**	**310 ± 0.000**	**590 ± 0.011**	**1.23**	**8.06 ± 0.02**	**-49 ± 3.11**	**< 2 μm**
7	4	284 ± 4.20	0.109 ± 0.037	300 ± 0.000	590 ± 0.000	1.00	8.02 ± 0.01	-52 ± 0.90	< 2 μm
	25	273 ± 2.90	0.125 ± 0.013	300 ± 0.005	580 ± 0.005	1.23	8.00 ± 0.02	-47 ± 0.51	< 2 μm
	40	276 ± 0.70	0.098 ± 0.017	310 ± 0.000	570 ± 0.005	1.23	7.99 ± 0.02	-46 ± 0.47	< 2 μm
14	4	277 ± 1.94	0.102 ± 0.030	300 ± 0.005	570 ± 0.025	1.23	8.01 ± 0.00	-46 ± 0.58	2–3 μm
	25	272 ± 1.77	0.128 ± 0.008	300 ± 0.000	580 ± 0.011	1.00	7.92 ± 0.01	-48 ± 0.60	2–3 μm
	40	278 ± 5.51	0.102 ± 0.011	310 ± 0.005	580 ± 0.011	1.23	7.93 ± 0.01	-47 ± 0.78	2–3 μm
30	4	275 ± 2.65	0.091 ± 0.056	300 ± 0.005	580 ± 0.001	1.00	7.98 ± 0.01	-49 ± 0.26	2–3 μm
	25	276 ± 2.33	0.088 ± 0.016	300 ± 0.011	580 ± 0.023	1.00	7.94 ± 0.00	-45 ± 0.50	2–3 μm
	40	233 ± 1.27	0.074 ± 0.042	290 ± 0.000	520 ± 0.011	1.00	7.69 ± 0.02	-39 ± 1.01	3–4 μm
**Lipofindin MCT/LCT**									
**0**	**4**	**260 ± 1.70**	**0.087 ± 0.013**	**310 ± 0.000**	**570 ± 0.000**	**1.23**	**7.54 ± 0.02**	**-39 ± 0.43**	**< 2 μm**
7	4	250 ± 1.56	0.087 ± 0.012	300 ± 0.005	570 ± 0.020	1.23	7.59 ± 0.01	-39 ± 0.50	< 2 μm
	25	256 ± 1.20	0.118 ± 0.021	310 ± 0.000	580 ± 0.005	1.23	7.55 ± 0.01	-40 ± 0.55	< 2 μm
	40	252 ± 1.64	0.088 ± 0.018	310 ± 0.000	580 ± 0.011	1.23	7.50 ± 0.00	-41 ± 0.28	2–3 μm
14	4	250 ±0.56	0.119 ± 0.021	300 ± 0.000	560 ± 0.005	1.23	7.54 ± 0.00	-43 ± 0.50	< 2 μm
	25	258 ±0.75	0.116 ± 0.021	300 ± 0.005	560 ± 0.011	1.23	7.52 ± 0.01	-41 ± 0.23	2–3 μm
	40	259 ±0.45	0.076 ± 0.023	300 ± 0.005	560 ± 0.017	1.23	7.48 ± 0.00	-39 ± 0.95	2–3 μm
30	4	250 ±1.06	0.078 ± 0.010	320 ± 0.000	580 ± 0.032	1.23	7.49 ± 0.02	-38 ± 0.98	< 2 μm
	25	254 ±1.06	0.083 ± 0.013	310 ± 0.011	580 ± 0.040	1.00	7.51 ± 0.02	-40 ± 0.55	2–3 μm
	40	224 ±2.04	0.097 ± 0.027	300 ± 0.000	530 ± 0.005	1.00	7.12 ± 0.00	-36 ± 0.25	3–4 μm
**ClinOleic**									
**0**	**4**	**256 ±2.30**	**0.119 ± 0.024**	**300 ± 0.000**	**560 ± 0.011**	**1.00**	**8.31 ± 0.01**	**-42 ± 0.60**	**< 2 μm**
7	4	265 ±0.70	0.109 ± 0.012	300 ± 0.005	570 ± 0.025	1.00	8.17 ± 0.02	-46 ± 0.90	< 2 μm
	25	252 ±0.88	0.098 ± 0.006	310 ± 0.005	550 ± 0.023	1.00	8.11 ± 0.01	-43 ± 0.10	< 2 μm
	40	253 ±2.38	0.099 ± 0.011	300 ± 0.005	550 ± 0.015	1.00	8.02 ± 0.02	-42 ± 0.43	< 2 μm
14	4	263 ±3.47	0.102 ± 0.015	310 ± 0.000	570 ± 0.011	1.23	8.13 ± 0.00	-48 ± 0.70	< 2 μm
	25	259 ±0.66	0.108 ± 0.011	300 ± 0.005	560 ± 0.012	1.23	8.09 ± 0.02	-47 ± 0.30	2–3 μm
	40	258 ±1.50	0.110 ± 0.015	300 ± 0.000	550 ± 0.005	1.00	8.01 ± 0.02	-44 ± 0.55	2–3 μm
30	4	262 ±2.56	0.119 ± 0.006	300 ± 0.005	560 ± 0.005	1.00	8.09 ± 0.01	-41 ± 0.70	< 2 μm
	25	255 ±1.82	0.105 ± 0.008	300 ± 0.000	560 ± 0.000	1.00	8.05 ± 0.00	-44 ± 0.41	2–3 μm
	40	239 ±3.21	0.093 ± 0.018	290 ± 0.005	540 ± 0.017	1.00	7.99 ± 0.01	-41 ± 0.68	3–4 μm
**SMOFlipid**									
**0**	**4**	**342 ±2.42**	**0.125 ± 0.017**	**340 ± 0.000**	**670 ± 0.005**	**1.23**	**6.83 ± 0.02**	**-56 ± 0.20**	**< 2 μm**
7	4	339 ±2.30	0.144 ± 0.026	320 ± 0.000	670 ± 0.000	1.23	6.87 ± 0.01	-58 ± 0.40	< 2 μm
	25	334 ±3.20	0.134 ± 0.024	330 ± 0.010	660 ± 0.026	1.23	6.80 ± 0.01	-48 ± 0.60	< 2 μm
	40	336 ±2.63	0.147 ± 0.012	330 ± 0.005	660 ± 0.015	1.23	6.79 ± 0.02	-52 ± 0.32	2–3 μm
14	4	334 ±2.65	0.125 ± 0.029	330 ± 0.000	650 ± 0.000	1.23	6.83 ± 0.00	-46 ± 0.51	< 2 μm
	25	335 ±2.90	0.144 ± 0.019	330 ± 0.011	650 ± 0.046	1.23	6.78 ± 0.00	-48 ± 0.88	2–3 μm
	40	331 ±3.56	0.135 ± 0.027	320 ± 0.000	670 ± 0.011	1.23	6.77 ± 0.02	-42 ± 0.05	2–3 μm
30	4	334 ±2.04	0.130 ± 0.023	320 ± 0.032	650 ± 0.010	1.23	6.79 ± 0.01	-47 ± 0.47	< 2 μm
	25	336 ±1.93	0.138 ± 0.021	330 ± 0.005	660 ± 0.015	1.23	6.76 ± 0.02	-48 ± 0.80	2–3 μm
	40	234 ±2.23	0.124 ± 0.024	300 ± 0.035	590 ± 0.020	1.23	6.40 ± 0.01	-38 ± 0.05	3–4 μm

Statistical significant differences (p<0.05), in compare with t = 0 for each emulsion, were observed only after 30 days at 40°C.

Lipid emulsions in the manufacturers’ containers were characterized by the size of the oil phase below 2 μm and the presence of 2–3 μm droplets. In all emulsions, after 30 days at 40°C, droplets of 2–3 μm were also observed (Fig 1, Table 2).

**Table 2:** Emulsions in manufacturer containers–the influence of time and temperature of storage (n = 9; mean ± SD; p< 0.05, t = 0 is presented in Table 1).

Time [days]	Temp[°C]	Size of oily droplets					pH	Zeta potential [mV]	Microscopic observation
		Z average [nm]	pDI	d_(0.5)_ [nm]	d_(0.9)_ [nm]	d_max_ [μm]			
**Omegaven**									
7	25	229±1.17	0.113±0.026	300 ± 0.005	550 ± 0.011	1.00	7.78 ± 0.01	-52 ± 0.10	< 2 μm
	40	233 ± 1.78	0.097 ± 0.010	300 ± 0.005	560 ± 0.030	1.00	7.68 ± 0.01	-55 ± 0.41	< 2 μm
14	25	227 ± 2.17	0.105 ± 0.019	300 ± 0.011	550 ± 0.030	1.23	7.69 ± 0.02	-52 ± 1.51	2–3 μm
	40	230 ± 2.09	0.102 ± 0.033	300 ± 0.000	560 ± 0.000	1.23	7.67 ± 0.00	-53 ± 0.43	2–3 μm
30	25	232 ± 2.07	0.103 ± 0.023	290 ± 0.005	560 ± 0.011	1.00	7.68 ± 0.01	-49 ± 0.40	< 2 μm
	40	242± 2.55	0.103 ± 0.030	310 ± 0.005	600 ± 0.035	1.23	7.30±0.01	-47 ± 0.69	3–4 μm
**Intralipid**									
7	25	276 ± 2.55	0.118 ± 0.020	310 ± 0.000	580 ± 0.011	1.23	7.89 ± 0.01	-53 ± 0.49	< 2 μm
	40	278 ± 1.37	0.129 ± 0.034	300 ± 0.000	570 ± 0.000	1.23	7.83 ± 0.02	-49 ± 1.10	2–3 μm
14	25	270 ± 0.60	0.107 ± 0.012	300 ± 0.005	580 ± 0.020	1.23	7.88 ± 0.02	-52 ± 0.65	2–3 μm
	40	274 ± 0.63	0.113 ± 0.008	310 ± 0.005	590 ± 0.150	1.23	7.81 ± 0.02	-49 ± 1.10	2–3 μm
30	25	271 ± 2.15	0.114 ± 0.001	310 ± 0.005	590 ± 0.020	1.23	7.83 ± 0.02	-50 ± 0.68	2–3 μm
	40	296± 0.15	0.129 ± 0.015	340 ± 0.005	680 ± 0.015	1.23	7.68±0.01	-47 ± 0.51	3–4 μm
**Lipofindin MCT/LCT**									
7	25	277 ± 1.55	0.127 ± 0.004	310 ± 0.005	560 ± 0.026	1.23	7.61 ± 0.03	-43 ± 0.80	< 2 μm
	40	278 ± 1.46	0.109 ± 0.004	300 ± 0.005	560 ± 0.037	1.23	7.56 ± 0.01	-45 ± 1.32	2–3 μm
14	25	283 ± 0.70	0.082 ± 0.025	300 ± 0.005	560 ± 0.015	1.23	7.58 ± 0.01	-44 ± 0.88	2–3 μm
	40	282 ± 2.27	0.100 ± 0.023	310 ± 0.005	570 ± 0.023	1.23	7.57 ± 0.02	-43 ± 1.12	2–3 μm
30	25	276 ± 1.71	0.095 ± 0.011	300 ± 0.005	560 ± 0.005	1.23	7.55 ± 0.02	-44 ± 0.45	2–3 μm
	40	241± 2.68	0.088 ± 0.018	320 ± 0.015	590 ± 0.026	1.23	7.43±0.02	-43 ± 0.51	3–4 μm
**ClinOleic**									
7	25	266 ± 0.37	0.090 ± 0.008	300 ± 0.005	570 ± 0.005	1.23	8.09 ± 0.00	-51 ± 0.70	< 2 μm
	40	264 ± 1.85	0.104 ± 0.007	300 ± 0.005	570 ± 0.011	1.00	7.95 ± 0.01	-53 ± 0.10	< 2 μm
14	25	267 ± 0.55	0.093 ± 0.017	290 ± 0.005	550 ± 0.015	1.00	8.06 ± 0.02	-48 ± 0.66	2–3 μm
	40	268 ± 1.07	0.111 ± 0.013	300 ± 0.005	550 ± 0.026	1.23	7.93 ± 0.01	-49 ± 0.25	2–3 μm
30	25	266 ± 1.32	0.124 ± 0.007	300 ± 0.005	560 ± 0.015	1.00	8.02 ± 0.00	-49 ± 0.65	< 2 μm
	40	271 ±1.99	0.112 ± 0.010	310 ± 0.005	570 ± 0.015	1.23	7.79±0.01	-44 ± 0.28	3–4 μm
**SMOFlipid**									
7	25	326 ± 2.45	0.125 ± 0.015	330 ± 0.000	630 ± 0.005	1.23	6.72 ± 0.00	-52 ± 0.95	2–3 μm
	40	325 ± 1.13	0.116 ± 0.017	330 ± 0.005	650 ± 0.000	1.23	6.60 ± 0.02	-53 ± 0.28	2–3 μm
14	25	326 ± 2.01	0.134 ± 0.018	340 ± 0.005	640 ± 0.037	1.23	6.69 ± 0.02	-47 ± 0.23	2–3 μm
	40	323 ± 2.52	0.106 ± 0.035	330 ± 0.040	650 ± 0.037	1.23	6.67 ± 0.02	-46 ± 0.26	2–3 μm
30	25	328 ± 1.90	0.129 ± 0.025	330 ± 0.005	650 ± 0.020	1.23	6.65 ± 0.02	-49 ± 0.30	2–3 μm
	40	333± 1.99	0.152 ± 0.024	360 ± 0.005	670 ± 0.015	1.23	6.52±0.01	-42 ± 0.90	3–4 μm

Statistical significant differences (p<0.05), in compare with t = 0 for each emulsion, were observed only after 30 days at 40°C.

### Measurement of the size of oil droplets

#### Laser diffraction method (LD)

The median diameter of oily droplets (d 0.5) of emulsions in polypropylene syringes ranged from 270 to 360 nm (Table 1). Omegaven was characterized by the smallest diameter of oil drops with a maximum value of 300 nm, while SMOFlipid showed the highest values of d0.5 (from 310 to 360 nm). The diameter of 90% of the oil phase drop of the emulsion in a plastic container was in the range of 470 to 670 nm. The same dependence was noticed, Omegaven was characterized by the lowest values (maximum 580 nm) and SMOFlipid the highest (from 590 to 670 nm). The maximum size of oily droplets does not exceed 1.23 μm. The median diameter of oil phase droplets (d0.5) for emulsions in the manufacturers’ containers was from 290 to 360 nm (Table 2). 90% of the oil droplets of the emulsion (d0.9) were from 520 to 680 nm. The lowest values of these parameters were characterized by Omegaven. The largest diameter of oil droplets was shown by SMOFlipid. The maximum drop size of the oil phase was not greater than 1.23 μm.

Statistically significant differences (p<0.05) in the size of oil droplets (a decrease for emulsions in polypropylene syringe and an increase for emulsion in the manufacturers’ containers) was noticed in all emulsions only after 30 days of storage at 40°C relative to results at time t = 0, but all emulsions were under USP limits.

#### Photon correlation spectroscopy (PCS) method

The Z-average parameter was in the range of 203 to 339 nm for all emulsions in polypropylene syringes (Table 1). Similar to the LD method, Omegaven was characterized by the smallest diameter of oil droplets regardless of packaging, temperature and time, while SMOFlipid was the largest (203 nm, 342 nm respectively). No increase in the size of oil droplets was observed after 30 days of storage at 4°C and 25°C. A statistically significant decrease (p <0.05) of the Z-average parameter for all emulsions after 30 days at 40°C was noticed (Table 1).

The oil droplet diameter of emulsions in commercial containers ranged from 229 to 333 nm (Table 2). The diameter of the droplets was the smallest for the Omegaven emulsion and the largest for the SMOFlipid. After 30 days at 40°C, the increase of oil droplets was statistically significant for all emulsions in the manufactured containers compared to t = 0. The statistical analysis (p <0.05) showed the effect of time and temperature on the Z-average parameter, regardless of the type of emulsion (Tables 1 and 2).

The analysis of variance showed differences in the size of oil droplets due to temperature (p < 0.05) and the study day (p < 0.05) but not the type of the emulsion (p = 0.98). The parenteral lipid emulsions in polypropylene syringe exhibited identical (except Z-avarage at 40°C, t = 30 days) time-dependent behavior with respect to mean globule size as compared to the original containers. The size of oily droplets of all test conditions remained below the United States Pharmacopeia limits.

### Zeta potential

All emulsions were characterized by a negative zeta potential. For the plastic, the zeta potential was in the range -36 mV to -58 mV (Table 1). The lowest negative zeta potential was found in Lipofundin LCT/MCT (-36 to -49 mV), the highest SMOFlipid (-42 to -58 mV). The zeta potential of emulsions in the manufacturers’ containers was -42 mV to -57 mV (Table 2). The lowest negative zeta potential was observed for Lipofundin, the highest for the Omegaven emulsion. The statistical analysis (p<0.05) showed statistically significant differences between the value of the emulsion potential at time t = 0 and t = 30, both for emulsions in polypropylene syringes and the manufacturers’ containers. In the case of emulsions in a plastic packaging, the differences were statistically significant for Intralipid and SMOFlipid at each temperature. For the Lipofundin and Omegaven emulsions, the differences were between 4°C and 25°C, for the ClinOleic 4°C and 40°C emulsions. For emulsions in PP syringes after 30 days, the negative potential decreased over time for Intralipid: -49 mV to -42 mV, Lipofundin formulation: -39 mV to -36 mV (25°C) and SMOFlipid: from -56 mV to -48 mV. For emulsions in the manufacturers’ containers after 30 days, an increase in the negative potential of Omegaven was observed: from -43 mV to -48 mV, Lipofundin: from -39 mV to -45 mV and ClinOleic: from -42 mV to -47 mV.

### pH measurement

The pH of emulsions were in the range of 6.40 to 8.31. The highest pH at t = 0 was noticed for the ClinOleic emulsion (8.31), the lowest for SMOFlipid (6.83). The pH of emulsions in original and polyproplyene containers decreased slightly during storage. Analysis of ANOVA variance (p <0.05) showed statistically significant differences (Tables 1 and 2) for emulsions after 30 days at 40°C with respect to t = 0, regardless of the type of container. There were no statistically significant differences for emulsions in PP syringes with respect to the original containers. This time-dependent pH decrease in the emulsion is independent of container type and may be a result of the production of free fatty acids after phospholipid hydrolysis in an aqueous system.

## Discussion

The stability of lipid injectable emulsions is a challenge in parenteral administration. Particularly in neonatology due to the small volume of the emulsion being administered and the possibility of its destabilization, the fat emulsion is separated from the other components by placing it in a polypropylene syringe. It is also possible that for patients in incubators, the emulsion will be exposed to temperatures above 30°C, which could affect its physicochemical stability. The compatibility of commercial lipid injectable emulsions transferred to polypropylene syringes and stored up to 30 days was examined. Of course, parenteral nutrition admixtures are administered only within 24 hours, but the aim of our study was to find the point when destabilization occurs. Five lipid parenteral emulsions commonly used in clinical practice were analyzed: Omegaven^,^ SMOFlipid, Intralipid, Lipofundin MCT/LCT, ClinOleic 20%. Significant parameters related to stability, including microscopic observation, pH and zeta potential measurement, size and distribution of oil globules were examined in the present study. The physiochemical properties of the emulsion in a polypropylene syringe were tested and compared to emulsions in original manufactures containers. The study was conducted at three different temperatures (4°C, 25°C, 40°C) for a period of 30 days to determine if temperature and time affect the stability of the emulsion.

Despite the fact that European and British Pharmacopeias contain no information about size of oily droplets in lipid emulsions, appropriate guidelines such as the United States Pharmacopeia with respect to lipid injectable emulsion globule size distribution (GSD) and percentage of fat residing in globules greater than 5 μm (PFAT5) is essential to improve the safe infusion of lipid emulsions across a variety of clinical uses. However, techniques based on light obscuration by individual globules are unable to accurately assess the GSD mean diameter, while globule sizes greater than about 2 μm in diameter can be accurately determined. The use of Coulter counters based on electrical pulse counting has been widely applied to the characterization of the larger globules in the GSD tail [12]. On the other hand, traditional particle sizing techniques based on light scattering (laser diffraction and dynamic light scattering) provide useful information about the mean size of the globule size distribution but are insensitive to changes in the GSD tail [13]. Microscopic observations are labor intensive and more susceptible to sampling errors and are subjective, so they are less likely to reflect the lipid globule size distributions of samples. Therefore, to determine the globule size distribution, at least two methods are necessary; one to determine the mean globule size and another to detect the possible presence of larger globules [12].

Visual observations throughout the study did not show any visible changes in fat emulsions, regardless of the temperature and the type of containers. No phase separation was observed. Microscopic observations showed an increased diameter of individual oil phase droplets (3–4 μm) for some emulsions stored at 40°C after at least 14 days. However, no increased number of large droplets was observed (Table 1).

The LD method did not confirm the significant increase of oil droplets, regardless of the time and storage temperature. The dmax parameter did not exceed 1.23 μm in all emulsions (Tables 1 and 2).

The PCS method allows measuring the average size of oil droplets up to 5 μm. For this reason, it is not the only method for evaluation of the stability of lipid emulsion. No increase of the Z-average parameter above 350 nm was noticed and all emulsions tested were monodispersed (Tables 1 and 2). The type of container has no influence on the stability of all emulsions. Values obtained by PCS were slightly (around 50 nm) lower than those obtained using the LD method. The statistical analysis did not show the effect of temperature on the stability of emulsions but showed significant differences in the Z-average parameter between t = 0 and t = 30 days.

All emulsions were characterized by a negative zeta potential. The temperature has no significant influence on the zeta potential parameter (Table 1). There were statistically significant differences in the value of zeta potential at t = 0 and t = 30 days. The pH values dropped slightly over time. Statistically significant differences (p <0.05) were noted over time for emulsions stored at 40°C (Tables 1 and 2). Lecithin is known to degrade on exposure to air, and for that degradation to cause a decrease in pH [14]. The extent of degradation and its effects are complicated and depends on the composition of the lecithin. If lecithin is dominated by phosphatidylcholine, released fee fatty acids partition into the oil phase and any decrease in pH is minimal. Faster degradation of lecithin and decreasing the pH occurs when phosphatidylethanolamine is dominated, due to release free fatty acids tend to partition into the aqueous phase [14]. In this study a similar pH decrease and a similar time scale, with ageing of the lecithin was noticed as a result of acid hydrolysis of the lecithin. So, there were no statistically significant differences between the injectable emulsions in the polypropylene and manufactures containers (Tables 1 and 2).

Considering the physical stability, all lipid injectable emulsions were stable for 30 days at 4°C and 25°C and only for 21 days at 40°C. Significant differences were noticed among lipid injectable emulsions after 30 days of storage in a polypropylene syringe at 40°C. All emulsions had a significant drop in the size of fat globules in contrast to the emulsion product in the manufacturers’ containers where increasing the size of fat globules was noticed. The reduction of the size of fat globules could be caused by the adsorption of larger oil droplets on the polypropylene surface and needs further analysis.

## Conclusions

All tested emulsions could be safely used in clinical practice on neonatal wards after repacking in polypropylene syringe. Considering the physical stability, lipid emulsions such as ClinOleic 20%, Intralipid, Lipofundin MCT/LCT, Omegaven and SMOFlipid can be safely stored after repacking to a polypropylene syringe for 30 days at 4°C and 25°C, however only 21 days when they are stored at 40°C. In clinical practice, longer storage of repacked emulsions exceeding 12 hours should be confirmed in a microbiological test. Additional measurements should be done to confirm that the large oil droplets are absorbed on the polypropylene surface of containers during exposure to the emulsion up to 30 days at 40°C.
